# Psychological distress in the workforce: a multilevel and longitudinal analysis of the case of regulated occupations in Canada

**DOI:** 10.1186/1471-2458-14-808

**Published:** 2014-08-07

**Authors:** Nathalie Cadieux, Alain Marchand

**Affiliations:** Department of Management and HRM, University of Sherbrooke, Sherbrooke, Canada; School of Industrial Relations, University of Montreal, Montreal, Canada

## Abstract

**Background:**

This study uses a multidimensional theoretical model to evaluate the role of regulated occupations and working conditions in explaining psychological distress.

**Methods:**

Various multilevel regression analyses were conducted on longitudinal data for which measures repeated over time (n_1_ = 36,166) were nested in individuals (n_2_ = 7007).

**Results:**

Results showed that when we controlled for working conditions, family situation, the social network outside the workplace, and personal characteristics, the level of psychological distress was significantly lower among professional workers in regulated occupations than among professionals not in regulated occupations. Among the working conditions studied, skill utilisation, psychological demands, and job insecurity were positively associated with psychological distress levels, whereas social support in the workplace was inversely related to distress. Finally, our results suggest that self-esteem reduced the effect of social support in the workplace on psychological distress levels in the workforce.

**Conclusions:**

These results support our hypothesis that working in regulated occupations exerts a direct effect on mental health. These results also make clear the importance of developing new tools for measuring psychological distress among upper-level professional workers. Such tools will be much better suited to the realities characterising today's knowledge-based economies.

## Background

In recent decades, the study of both workplace mental health and the mechanisms underlying the development or worsening of mental health problems has assumed a prominent place in the literature. This may be explained by the extensive costs that organisations have incurred from absenteeism, turnover, and enhancements to productivity, among other factors [1–3]. In Canada, every day, 500,000 workers are absent from work due to mental health problems, which represents an economic burden estimated to exceed Cdn$30 billion per year [4]. In the United States, the costs associated with workplace stress reportedly come to nearly $200 billion annually [5]. In Europe, a recent study put the cost of stress in France for 2007 at between €2 billion and €3 billion [6, 7], and losses from workplace stress in 2002 reached nearly €20 billion for the EU-15 [6, 8].

Although several studies have examined the role of occupations and the workplace [9–12], the study of mental health among workers in regulated occupations has not received much attention to date. Regulated occupations are those occupations in which the practices and titles are legally determined by professional associations responsible for protecting the public, particularly through the issuance of licenses to those with the education and skills required to practice regulated occupations. For example, these professions include doctors, midwives, engineers, lawyers, nurses, dentists, pharmacists, etc.

These regulated occupations constitute a closed labour market and share some characteristics specified by the legislature in justifying the supervision of such occupations by the professional associations responsible for protecting the public. These conditions pertain to: (1) the knowledge required to engage in the activities of the persons who would be governed by the proposed order; (2) the degree of independence enjoyed by the persons who would be members of the order when engaging in the activities concerned, and the difficulty which persons not having the same training and qualifications would have in assessing these activities; (3) the personal nature of the relationships between such persons and those having recourse to their services, by reason of the special trust which the latter must place in them, particularly because such persons provide them with care or administer their property; (4) the gravity of the prejudice which might be sustained by those who have recourse to the services of such persons if their competence or integrity was not supervised by the order; (5) the confidential nature of the information which such persons are called upon to access while practising their profession [13].

These characteristics define a body of working conditions shared by regulated occupations which can constitute stressors in the work environment in which professionals exercise their professions and act as constraints for them.

As revealed by the literature, the functionalist school [14], the interactionist school [15, 16] and the monopolistic school [17] developed an important body of arguments regarding the characteristics shared by regulated occupations [14]. For example, by virtue of their status, the professionals working in regulated occupations enjoy significant autonomy in the execution of their work. This leads to an inability on the part of employers to control their work processes. Conversely, while such autonomy may in itself be a protective factor for the psychological health of these professionals, it remains that the reverse side of autonomy is responsibility. Professionals are independent but accountable.

Several theoretical arguments have been put forward supporting the validity of considering regulated occupations as an occupational group in the study of psychological distress.

In empirical terms, apart from statistics published annually by some professional associations, we have little basis for making comparisons. Is the level of psychological distress among regulated occupations really higher than that for other socio-occupational categories? Whatever the answer to this question, what working conditions might explain any such differences?

This study seeks to identify the specific contributions of regulated occupations and the workplace to psychological distress by using a model that takes into account individual characteristics, family, and the social network outside the workplace. The working conditions considered include skill utilisation, decision authority, psychological demands, physical demands, social support, job insecurity, hours worked and work-schedule irregularity. Our analyses are based on longitudinal data gathered at seven points in time between 1994–1995 and 2006–2007.

### Theoretical model and hypotheses

Too often the study of mental health fails to consider the contribution that social dimensions make to explanations of the development and intensification of mental health problems [18, 19].

The multilevel model of mental health determinants in the workforce [19, 20] takes as its general hypothesis that mental health problems that workers experience (e.g., psychological distress) result from stress. This stress is, in turn, attributable to the constraints and resources simultaneously brought to bear simultaneously by agent personality (the microsocial level: demographics, health, life habits, psychological traits, stressful childhood events), structures of daily life (the mesosocial level: workplace, family, social network outside the workplace), and macrosocial structure: economic and occupational structure, policies, culture). This model is based on a set of theoretical postulates. First, the model takes a page from micro and macro frameworks in sociology [21, 22] and from the agent-structure approach [23, 24]. These postulate that the catalyst for action resides in social structures and that this action is also influenced by both the power and the skills of actor-agents. Second, the model is also influenced by sociological theory of stress [25] in which constraints are stressors that have the potential to affect an individual’s adaptability. It enables us to explain how psychological distress arises in the workforce by, on the one hand, bringing in the specific contribution of the workplace and, on the other hand, measuring the contribution of non-work and family-related factors, as well as factors linked to individual characteristics. The model generates three main hypotheses.

H1: Regulated occupations, working conditions, family, the social network outside the workplace, and individual characteristics contribute directly and jointly to explaining the level of psychological distress.

This first hypothesis postulates the existence of a direct link between the level of psychological distress and the practice of a regulated occupation. It represents an original contribution to our understanding of the relationship between mental health and regulated occupations. Current research, which looks at the role played by certain regulated occupations in the development of mental health problems [9–12, 26], has generally proceeded in a somewhat segmented fashion by examining these occupations individually rather than as a group subject to statutory controls over occupational practice and titles. Even when a comparison is made of the levels of stress among most categories of workers [27], the emphasis has not been placed on the fact that one of the causes of the observed differences may be the presence or absence of regulatory rules.

This hypothesis also presupposes a direct link between the constraints and resources engendered by working conditions and psychological distress levels. The direct relationship between working conditions and psychological distress is conditioned by theoretical models of occupational stress [28–30] and by the empirical results obtained from applying them in a variety of work settings. According to the demand-control model [28], high levels of demand combined with low levels of control increase the risk of experiencing mental health problems. This hypothesis has received considerable empirical support [9, 11, 31, 32]. The effort-reward imbalance model [30], for its part, has contributed a framework for understanding the mechanisms underlying the perceptual effects of working conditions. It postulates that the perception of imbalance between effort made by workers and rewards received leads to mental health problems [30]. This hypothesis has also received considerable support in the literature [11, 32, 33].

These models make it possible to identify a number of workplace factors that are related to the level of psychological distress. Control (in the broad sense), skill utilisation, task variety, social support in the workplace, and rewards are thought to be associated with lower levels of psychological distress [2, 9, 32–34]. Conversely, the demands to which individuals are exposed in work settings represent constraints associated with higher levels of psychological distress [35, 36].

Hypothesis 1 also assumes the existence of a direct relationship between psychological distress and both the family and the social network outside the workplace. Not having a partner or living alone, like stresses in marital and parental relationships, has been associated with higher levels of psychological distress [31, 37]. Conversely, being the parent of young children (0 to 5 years old), having a high family income, and having access to a social network outside the workplace are associated with lower levels of psychological distress [31, 38, 39].

Finally, research has shown psychological distress levels to be directly associated with individual personal characteristics. The literature generally reports psychological distress levels as being higher among women [40, 41]. Excessive alcohol consumption, smoking, and stressful childhood events also contribute to psychological distress [42]. Conversely, psychological distress levels tend to diminish as age increases and are lower among people who have high self-esteem [39, 43], an internal locus of control [44], and a strong sense of cohesion, as well as among immigrants [45–47].

H2: The workplace mediates the relationship between regulated occupations and psychological distress levels.

The second research hypothesis posits that the workplace mediates the relationship between regulated occupations and psychological distress levels. This means that each regulated occupation generates working conditions that are specific to it [9–12, 26, 44, 48–50]. Task design seems to constitute a resource rather than a constraint in the regulated occupations. Working conditions in these occupations are characterised by a certain level of control, a degree of decision authority, and considerable skill utilisation, as well as by varied tasks—characteristics that in themselves constitute protective mental health factors [2, 33, 34, 51]. This should also be the case for the gratifications that professional workers derive from working in the regulated occupations, which offer a certain prestige, remuneration levels that are higher than the average for non-regulated occupations and a degree of job security [17]. These factors, linked as they are to rewards and being associated with lower levels of psychological distress [9, 11, 32, 52], should constitute a resource for professional workers in the regulated occupations.

Conversely, the demands associated with these occupations present risk factors for the mental health of professional workers [9, 44, 49, 53]. Contract-related demands, such as the number of hours worked [9], and psychological demands—including heavy workloads, the fear of committing malpractice, client expectations, clerical and administrative tasks, budgetary pressures, role-generated stress (e.g., conflict, ambiguity, overwork)—may contribute to the experience of stress among professional workers [9, 44, 49, 53]. Finally, with regard to social relationships, some professional workers in the regulated occupations complain of a lack of both feedback and support from supervisors, which is associated with higher levels of psychological distress [11, 54].

H3: The relationship between the workplace and psychological distress levels is moderated by individual characteristics, as well as by family and the social network outside the workplace.

The literature suggests that living with a partner or having young children, and having a high family income, as well as access to a social network outside the workplace, help attenuate the impact of certain stressors [37, 39, 55]. By contrast, marital and parental stress reduces the availability of resources to individuals and affects their ability to deal with workplace-related constraints [31]. For example, individuals facing a separation or divorce, or tensions in their relationship with their child or teenager, are already exposed to some level of stress. Their resources are already mobilised to deal with these challenges of everyday life. These individuals therefore have fewer resources to overcome stressors emanating from the workplace.

The same holds true for individual personal characteristics. For instance, the existence of a gender gap heightens the negative effect of certain working conditions, including work schedules, less control over work, greater effort to meet work demands, and emotional involvement [37, 56], which in turn leads to higher levels of psychological distress. This is explained by the tendency of women to report their symptoms more than men. It is therefore possible that exposure to certain working conditions is also more often reported by women as stressful, contributing to a higher level of psychological distress.

Age also moderates the relationship between working conditions and perceived stress levels, although the impact of workplace stressors generally decreases with age [9, 40]. More specifically, this relationship can be explained by the experience of older workers, which enables them to do the same work using fewer resources than young people while the stressors of life for older workers also tend to be less pronounced. Indeed, for this category of workers whose children have generally reached adulthood, the financial burden associated with the family is reduced, as are the resources mobilised for the family in general. Conversely, younger workers are more likely to have young children and a greater financial burden; they are accordingly in a period of life where everything is under construction. This mobilisation of resources, which is added to the tensions emerging from the beginning of their careers, may explain why the tensions generated by certain working conditions can be more pronounced for younger workers, who are also generally less experienced and more likely to experience psychological distress than older workers [9, 40].

Certain lifestyle habits, including physical activity [50], and certain personality traits, such as internal locus of control [44, 57] and sense of cohesion [58], also contribute to lowering the negative impact of certain work-related stressors and thus to reducing psychological distress levels in the workforce. Last, having immigrant status may also moderate the impact of certain working conditions via a "healthy immigrant" effect [45–47]. By controlling for certain sociodemographic characteristics such as age and gender, some studies have shown that, in general, immigrants actually enjoy better mental health than do native-born Canadians [45–47]. This effect, however, is known to diminish over time [45–47].

## Methods

### Data

Our study uses data from the first seven cycles of Statistics Canada's National Population Health Survey (NPHS) (Cycle 1: 1994–1995; Cycle 7: 2006–2007). Every two years the NPHS gathered longitudinal data on the health of Canadians for a very broad, representative sample of the population.

The initial NPHS sample comprising 17,276 persons in Cycle 1 was obtained using a stratified, two-stage sampling design (clusters and dwellings). The first stage allowed us to derive homogeneous strata from the Canadian provinces in order to take independent cluster samples in each of the strata. The second stage entailed selecting a certain number of households from the list of households (dwellings) for each cluster, and then choosing at random a household member to serve as the longitudinal respondent. The response rates from Cycle 1 through Cycle 7 ranged from 77.0% to 93.6%. Data were weighted by taking into account, first, the probabilities of selection and of non-response for each survey cycle. Second, weights in each province were post-stratified by age and sex using population estimates from the 1996 census. After eliminating missing values and selecting only employed persons aged 20 to 75 years, the weighted sample comprised 276 respondents working in regulated occupations and 6731 in non-regulated occupations.

### Measures

#### Psychological distress

Psychological distress was measured in the NPHS using the K6 scale [59], which measures non-specific psychological distress. Respondents evaluated six items on a 5-point additive scale (always/never), indicating how often during the preceding month they had had certain symptoms. These values yielded a global psychological distress score between 0 and 24 (alpha = 0.77). Because the distribution was asymmetric, a square-root transformation was applied to obtain a normal distribution so as to ensure a better fit with the multivariate analysis postulates [60]. After transformation, the psychological distress scale ranged from 0 to 4.9.

#### Occupation

Occupation was measured using the four-digit codes from the Standard Occupational Classification (SOC-1991) of Statistics Canada. In all, 471 occupations were first classified into 16 categories derived from the classification scheme of Pineo et al. [61], which classifies occupations by practice conditions that have comparable prestige, salary or wages, and educational requirements. In order to take into consideration the large number of categories, as well as the increased risk of developing mental health problems in certain occupations, these 16 categories were next combined into six large occupational groups: executives, managers, supervisors, professional workers, white-collar workers, blue-collar workers. These groups, which had been used in previous Canadian studies [57, 62], were comparable to those used in the United Kingdom. The seventh category comprised the regulated occupations under study here. It took as its point of reference the 25 regulated occupations in Québec.

A comparative interprovincial analysis was carried out based on these 25 regulated occupations to determine which ones met the regulated-occupation criteria in which provinces. Following this comparative analysis, a systematic selection procedure was applied. We eliminated all occupations for which the SOC-1991 codes included other occupational titles, some of which did not belong to regulated occupations (n = 8). These eight occupations were thus included in categories 1 through 6, but not in category 7 (regulated occupations). The occupation of notary, which exists only in Québec, was grouped with lawyers because of similarities in educational preparation and practice conditions. In this way we identified 17 occupations whose impact on individual mental health we could evaluate across all Canadian provinces. These regulated occupations are: architect, chemist, chiropractor, dentist, denturist, engineer, geologist, land surveyor, lawyer, medical radiation technologist, notary, nurse, optician, optometrist, pharmacist, physician (including specialist) and veterinarian. Since the regulations applying to regulated occupations vary by province, recoding allowed us to separate regulated from unregulated occupations in each province, where 1 = regulated occupation and 0 = unregulated occupation. The unregulated occupations were then reclassified into separate categories (executives, managers, supervisors, professional workers, white-collar workers, blue-collar workers).

#### Working conditions

Skill utilisation, decision authority, physical and psychological demands, social support, and job insecurity were measured in cycles 1 and 4 through 7. They came from Statistics Canada’s modified version of the Job Content Questionnaire [63]. Responses were based on a five-point Likert scale (strongly disagree-strongly agree). Skill utilisation was measured by three items (alpha = 0.53). Decision authority included two items (alpha = 0.65). Physical demands were measured by one item and psychological demands by two items (alpha = 0.35). Social support was measured by three items (alpha = 0.42) and job insecurity by one item. Contractual demands were evaluated using two items measured in cycles 1 through 7 (number of hours worked per week in all jobs including overtime; work-schedule irregularity using an eight-point scale where 0 = normal shift, 1 = rotating, broken, on-call, other).

#### Personal characteristics

Gender was measured with a dichotomous variable (0 = male; 1 = female). Age corresponded to the age reported by the respondent, in years. Immigrant status was measured using a dichotomous variable (0 = non-immigrant; 1 = immigrant). Self-esteem and locus of control were measured in cycles 1 and 4 through 7 using an additive five-point scale (strongly disagree/strongly agree). Six items [64] were used to measure self-esteem (alpha = 0.85). Seven items [65] were used to measure locus of control (alpha = 0.76). Sense of cohesion was measured by a 7-point additive scale (which varied for each of 13 items) [66] (alpha = 0.83). Lifestyle habits were measured in cycles 1 through 7. Alcohol consumption was expressed by the number of glasses consumed during the preceding week; smoking by the number of cigarettes smoked per week; and physical activity by the frequency of engaging in at least one activity longer than 15 minutes during the preceding three months. Stressful childhood events were evaluated in cycles 1, 4, and 7 via 7 items using a yes/no dichotomous variable [67] (alpha = 0.54). These events refer to a divorce or separation of parents, drug or alcohol abuse by a parent, an event which frightened the individual in childhood, etc.

#### Family

Marital status was measured in cycles 1 through 7 using a dichotomous variable (1 = married or civil union; 0 = other). Parental status was measured in cycles 1 through 7 with 3 dichotomous variables (1 = present, 0 = absent) indicating the presence or absence of children in the household in the following age groups: 0–5 years, 6–11 years, and 12–24 years. Marital stress and parental stress were measured in cycles 1 and 4 through 7 using an additive dichotomous variable (1 = true; 0 = false). Marital stress was measured using three items; parental stress, using two items [67]. Household economic status was measured for cycles 1 through 7 using an ordinal scale consisting of five categories (1 = low income to 5 = upper income) that measured income sufficiency as calculated by Statistics Canada, taking into account the number of persons in each household.

#### Social support outside the workplace

Social support outside the workplace was measured using an additive five-point (never/always) scale for all three items. Because of its asymmetry, the scale was reduced to two categories: low (0 = 0, 1, 2) and high social support (1 = 3, 4).

### Analysis

Multilevel multiple regression analyses, corrected for design effects, were used to analyse and compare the level of psychological distress across the seven cycles of the NPHS (a 13-year period) by occupation practised, working conditions, personal characteristics, family situation, and social network. The dataset had a hierarchical structure in which time (n_1_ = 36,166) was nested in individuals (n_2_ = 7007). If we assume a random distribution for missing values, respondents who did not participate in all cycles of the survey remain in the sample but contribute less than other respondents to explaining variations in psychological distress over time. The models were estimated using MlwiN statistical software, version 2.23. Descriptive analyses were performed using Stata software.

For these analyses, data were weighted using the bootstrap weights specified in the NPHS so as to take into account design effects generated by this type of survey. For multilevel analyses, model parameters were estimated using the iterative generalised least-squares (IGLS) method [68]. The significance of regression coefficients at the individual level was evaluated by performing a bilateral Z test (p ≤ 0.05). For the random part of the model, Wald tests were performed by plotting the value of p divided by 2 (p ≤ 0.05) [69]. The significance of the entire model was also evaluated with a Wald test (p ≤ 0.05). Because the data were weighted, "sandwich" standard errors were estimated to account for design effects generated by the complex sampling design of the NPHS. Standard errors were then corrected using the estimated design effect for Cycle 1 of the NPHS. This procedure involves inflating standard error estimates by the square root of the NPHS design effect. This method has been used with success in previous studies [20, 38, 57].

## Results

Table 1 presents statistics describing the entire sample from Cycle 1 through Cycle 7 (1994–2007) of the NPHS.

**Table 1:** **Sample descriptive statistics, NPHS, cycles 1 through 7**

	Cycle 1		Cycle 2		Cycle 3		Cycle 4		Cycle 5		Cycle 6		Cycle 7	
	N = 7007		N = 6163		N = 5610		N = 5165		N = 4529		N = 4128		N = 3564	
	Avg.	SD	Avg.	SD	Avg.	SD	Avg.	SD	Avg.	SD	Avg.	SD	Avg.	SD
**Mental health**														
Psychological distress	1.52	1.67	1.24	1.57	1.25	1.50	1.05	1.44	1.14	1.35	1.08	1.28	1.06	1.19
(square root)														
**Occupations (%)**														
Executives	0.56	-	0.46	-	0.41	-	0.56	-	0.68	-	0.70	-	0.46	-
Managers	7.58	-	7.52	-	8.20	-	10.16	-	10.73	-	11.67	-	12.20	-
Supervisors														
-	4.53	-	5.02	-	4.69	-	6.30	-	6.28	-	5.10	-	4.43	-
Professional workers	12.41	-	13.38	-	13.94	-	15.98	-	18.13	-	18.24	-	19.57	-
White-collar workers	47.24	-	45.52	-	44.51	-	39.24	-	38.30	-	37.77	-	37.60	-
Blue-collar workers	23.41	-	23.47	-	23.44	-	22.30	-	20.14	-	20.94	-	19.50	-
Regulated occupations	4.27	-	4.63	-	4.81	-	5.46	-	5.74	-	5.58	-	6.24	-
**Work conditions**														
Skill utilisation	7.12	3.35	7.23	3.14	7.27	3.00	7.34	2.87	7.34	2.69	7.43	2.57	7.46	2.39
Decision authority	5.44	2.51	5.48	2.36	5.47	2.25	5.50	2.16	5.55	2.02	5.62	1.93	5.59	1.79
Psychological demands	4.50	2.51	4.55	2.36	4.58	2.25	4.50	2.16	4.50	2.69	4.52	1.93	4.51	2.39
Physical demands	2.04	1.67	2.03	1.57	2.03	1.50	1.82	1.44	1.77	1.35	1.77	1.93	1.73	1.79
Social support	7.96	2.51	7.98	2.36	7.98	3.00	7.93	2.16	7.91	2.69	7.99	2.57	7.96	2.39
Job insecurity	1.54	1.67	1.50	1.57	1.52	1.50	1.27	1.44	1.31	1.35	1.37	1.28	1.36	1.19
Hours worked	42.90	23.44	44.14	22.77	44.52	21.72	42.16	17.25	41.82	16.82	41.92	16.06	41.62	17.31
Work-schedule irreg. (%)	21.36	-	20.21	-	20.76	-	18.66	-	18.25	-	19.78	-	18.33	-
**Personal characteristics**														
Gender (% female)	47.48	-	45.88	-	45.66	-	45.44	-	45.58	-	46.74	-	46.55	-
Age	38.48	11.72	39.84	11.78	41.40	10.49	43.20	10.78	44.81	10.77	46.35	10.92	48.01	10.75
Smoking	5.29	12.56	4.86	11.78	4.47	10.49	3.74	10.06	2.98	9.42	2.93	9.64	2.51	8.36
Alcohol consumption	3.68	10.04	3.54	10.21	3.78	9.74	3.50	7.91	3.71	10.09	3.77	10.92	4.41	14.92
Physical exercise	19.26	30.13	20.59	29.05	22.36	28.46	19.92	23.72	23.62	27.59	23.25	26.34	27.50	30.45
Self-esteem	20.46	4.19	20.48	3.93	20.46	3.74	19.92	3.59	19.91	4.04	19.89	3.85	19.97	3.58
Sense of cohesion	59.00	15.07	59.04	15.70	62.45	13.48	62.56	14.37	62.63	14.13	62.59	14.13	62.79	13.73
Locus of control	20.11	5.86	20.23	5.50	20.27	5.99	20.34	4.31	19.99	5.38	20.17	5.14	20.26	5.37
Stressful childhood events	0.55	0.84	0.57	1.57	0.60	1.50	0.64	1.44	0.68	1.35	0.72	1.28	0.78	1.19
Immigrant (% immigrant)	18.36	-	17.55	-	17.09	-	16.94	-	15.88	-	15.45	-	15.18	-
**Family**														
Marital status (% in couple)	71.15	-	71.40	-	72.57	-	74.27	-	75.30	-	76.57	-	76.01	-
Children 0–5 years (%)	22.55	-	20.86	-	20.29	-	17.11	-	15.15	-	12.47	-	11.01	-
Children 6–11 years (%)	21.07	-	22.42	-	23.17	-	24.43	-	22.88	-	22.66	-	20.54	-
Children 12–24 years (%)	26.26	-	25.70	-	25.46	-	26.54	-	28.18	-	30.43	-	31.72	-
Household income sufficiency	3.69	0.67	3.79	0.79	4.02	1.50	4.18	1.44	4.29	0.67	4.38	1.28	4.51	1.19
Marital stress	0.22	0.84	0.20	0.79	0.19	0.75	0.16	0.72	0.18	0.67	0.17	0.64	0.18	0.59
Parental stress	0.31	0.84	0.31	0.79	0.31	0.75	0.30	0.72	0.33	0.67	0.34	0.64	0.34	1.19
**Social network outside work**														
Social support (% high)	84.55	-	87.83	-	92.15	-	93.23	-	93.77	-	94.23	-	94.00	-

Table 2 presents the results of the eight estimated multilevel multiple regression models.

**Table 2:** **Multilevel multiple regression analyses**

	Model 1	Model 2	Model 3	Model 4	Model 5	Model 6	Model 7	Model 8
***Constant***	1.513**	1.453**	1.626**	1.517**	3.780**	1.710**	1.799**	3.937**
***Point in time***								
Cycle 2	-.291**	-.290**	-.291**	-.291**	-.274**	-.281**	-.281**	-.261**
Cycle 3	-.266**	-.264**	-.267**	-.266**	-.144**	-.242**	-.245**	-.122**
Cycle 4	-.467**	-.462**	-.448**	-.442**	-.308**	-.408**	-.416**	-.277**
Cycle 5	-.419**	-.416**	-.402**	-.396**	-.257**	-.364**	-.370**	-.226**
Cycle 6	-.456**	-.453**	-.437**	-.431**	-.272**	-.394**	-.405**	-.237**
Cycle 7	-.489**	-.486**	-.469**	-.463**	-.287**	-.424**	-.438**	-.249**
***Occupation***								
Regulated occupations (ref)	-	-	-	-	-	-	-	-
Executives		-.031		-.004	.061	-.003	-.011	.060
Managers		.023		.041	.032	.027	.037	.023
Supervisors		.008		.022	.012	-.001	.014	-.005
Professional workers		.088*		.116**	.108**	.105**	.115**	.102**
White-collar workers		.170**		.131**	.064	.100**	.128**	.049
Blue-collar workers		-.016		.002	-.018	-.023	-.013	-.034
***Work conditions***								
Skill utilisation			.001	.002	.015**	.004	-.002	.015**
Decision authority			-.022**	-.021**	.005	-.019**	-.021**	.006
Psychological demands			.037**	.037**	.023**	.036**	.037**	.023**
Physical demands			.002	.007	-.001	.003	.007	-.003
Social support			-.037**	-.037**	-.019**	-.035**	-.035**	-.017**
Job insecurity			.059**	.060**	.028**	.054**	.058**	.026**
Hours worked			.000	.001	.001	.001	.001	.001
Work-schedule irregularity			.019	.020	.015	.017	.020	.013
***Personal characteristics***								
Gender (female)					.158**			.157**
Age					-.009**			-.011**
Smoking					.004**			.003*
Alcohol consumption					.004**			.004**
Physical exercise					.000			.000
Self-esteem					.002			.002
Sense of cohesion					-.024**			-.022**
Locus of control					-.044**			-.040**
Stressful childhood events					.069**			.062**
Immigrant					-.022			-.029
***Family***								
Marital status (in couple)						-.224**		-.079**
Children 0–5 years						.002		-.045**
Children 6–11 years						-.010		-.032
Children 12–24 years						-.004		-.009
Household income sufficiency						-.037**		-.010
Marital stress						.179**		.089**
Parental stress						.131**		.093**
***Social network outside work***								
Social support (high)							-.349**	-.241**
***Random part of the model***								
σ^2^ Time	.593**	.593**	.590**	.590**	.573**	.584**	.586**	.565**
σ^2^ Individuals	.396**	.390**	.362**	.356**	.191**	.327**	.342**	.186**
Time (Chi-square)(6 df)	1436.1**	1405.1**	1318.9**	1279.4**	665.7**	1040.4**	1137.8**	558.7**
Individuals (Chi-square)(1 df)	2199.9**	2193.9**	2064.8**	2057.4**	1361.3**	1992.4**	2054.4**	1360.5**
Occupation (Chi-square)(6 df)	-	64.1**	-	74.3**	43.4**	68.6**	85.4**	47.0**
***Adjustment***								
Chi-square	-	1500.8**	2125.1**	2210.5**	7722.3**	2951.3**	2638.0**	8567.5**
(df)		(12)	(14)	(20)	(30)	(27)	(21)	(38)
R^2^ Time	.031	.037	.068	.074	.252	.11	.091	.265
R^2^ Individuals	.013	.027	.084	.096	.440	.160	.126	.452

*p ≤ 0.05 **p ≤ 0.01.

Model 1, which evaluates the link between the level of psychological distress and time, suggests that psychological distress levels diminished across the seven cycles and that the reduction was statistically significant (p ≤ 0.01). Model 2 estimates the effects of occupation and time on psychological distress levels. This model shows that the level of psychological distress was significantly higher among professional workers (p ≤ 0.05) and white-collar workers (p ≤ 0.01) than among professional workers in regulated occupations. Model 3 evaluates the effects of working conditions on the level of psychological distress and shows that decision authority, psychological demands, social support in the workplace, and job insecurity were statistically significant for explaining psychological distress levels for the sample (p ≤ 0.01). Model 4, when compared with Model 2, shows that even after controlling for working conditions, psychological distress levels were significantly higher among professional workers and white-collar workers and that this difference was statistically significant (p ≤ 0.01). Moreover, the likelihood of rejecting the null hypothesis when true decreased from p ≤ 0.05 to p ≤ 0.01 for the professional worker category.

Model 5 evaluates the effect of personal characteristics on the level of psychological distress by controlling for working conditions, occupation, and time. This model shows that gender, age, smoking, alcohol consumption, sense of cohesion, locus of control, and stressful childhood events were statistically significant for explaining the level of psychological distress (p ≤ 0.01). By contrast with Model 4, controlling for personal characteristics eliminated two effects. First, decision authority lost statistical significance for explaining psychological distress. Second, the difference in distress between white-collar workers and professional workers in regulated occupations was also no longer statistically significant. Conversely, whereas the level of skill utilisation was not statistically significant in models 3 and 4, Model 5 suggested that skill utilisation levels became statistically significant for explaining psychological distress when effects related to individual personal characteristics were controlled for.

Model 6 evaluates the effect of family on the level of psychological distress by controlling for time, occupation, and working conditions. It shows the level of psychological distress to have a statistically significant association (p ≤ 0.01) with marital status, household income sufficiency, marital stress, and parental stress. Model 7 evaluates the effect of the social network outside the workplace on psychological distress levels by controlling for time, occupation, and working conditions. It shows that high levels of social support have a statistically significant negative association with psychological distress levels (p ≤ 0.01).

Model 8 evaluates the effects of all variables on psychological distress levels. It is evident from this model, as was the case for Model 5 in which personal characteristics were controlled for, that differences in psychological distress levels between white-collar workers and professional workers in regulated occupations disappeared, whereas the effects related to working conditions changed. Thus, decision authority lost statistical significance whereas, conversely, psychological distress levels showed a statistically significant association (p ≤ 0.01) with skill utilisation levels.

**Figure 1:**
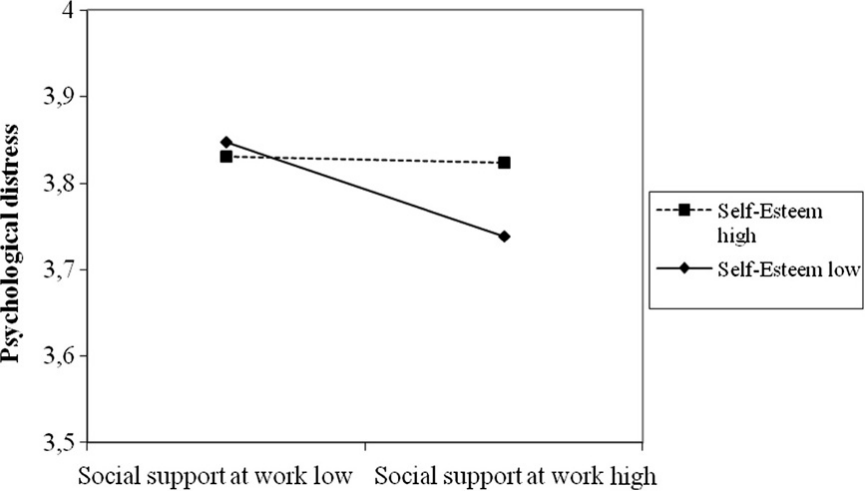
**Interaction between social support in the workplace and self-esteem.**

Last, we performed separate tests on the interactions between working conditions and personal characteristics, family, and the social network. Two interactions proved statistically significant: the interaction between income sufficiency and job insecurity (X^2^ = 31.26, p ≤ 0.01) and the interaction of social support in the workplace with self-esteem (X^2^ = 24.29, p ≤ 0.01). When evaluated together in Model 7 (X^2^ = 29.8, df 2, p ≤ 0.01), only the interaction between social support in the workplace and self-esteem remained (γ_interaction_ = 0.003, p ≤ 0.05). Figure 1 shows a decrease in the influence of high social support at work on the reduction of psychological distress when the self-esteem of an individual was high.

## Discussion

The results of this study support our hypothesis that regulated occupations and certain working conditions make specific contributions to explanations for variations in psychological distress over time (H1). These contributions exist independently of individual personal characteristics, family situation, and the existence of a social network outside the workplace.

It should also be noted that the influence of working conditions on psychological distress levels remains relatively stable when we control for occupation. Overall, the results suggest that it is important to consider all dimensions of the lives of individuals if we are to understand how mental health problems develop or intensify, as the literature has highlighted in recent years [3, 18–20, 38, 50].

First, psychological distress levels appear to have diminished across the seven cycles of the NPHS. This decline is statistically significant (p ≤ 0.01). These results could be explained by the evolution of the age of individuals across the cycles and by the protective effect of experience, which allows individuals to be more confident in their functions while reducing the effect of certain constraints tied to their working conditions. Aged workers may be allow for more work control because of their increased experience. Also, workers’ life conditions improved over time and their feelings of stress decreased [38]. These results are, moreover, consistent with those obtained in previous studies on mental health in the Canadian workforce and carried out with NPHS data [20, 57].

The contribution of occupation to psychological distress at work remains low. For instance, when we consider all aspects of an individual's life, the variation in psychological distress explained by occupation, based on changes in R^2^_individuals_ reported in Table 2, rises to 1.4%. Only the professional worker category may be distinguished in a significant way from professional workers in the regulated occupations. These results are in line with other results published earlier based on longitudinal data from the NPHS, showing that one’s position in the occupational structure explained 1.6% of the variation in psychological distress [20]. Although the percentage is small, the fact that a statistically significant distinction exists between professional workers in the regulated occupations and the professional worker category supports the assertion of the first hypothesis (H1) that occupation exerts a direct effect on individual psychological distress levels. This result highlights the direct role of regulated occupations in explaining psychological distress, even after controlling for the specific context in terms of the working conditions of a given professional practice. Regarding this result, it seems possible that the regulatory rules governing the exercise of regulated occupations contribute to mental health. Further research is needed to study the impact of this regulatory framework on the level of psychological distress among professionals.

A direct effect is also seen for certain working conditions where skill utilisation, psychological demands, social support in the workplace, and job insecurity contribute directly and in statistically significant ways to the level of psychological distress. Working conditions explain 3.7% of the variation in psychological distress over time and 7.1% of the variation among individuals. Thus, skill utilisation, psychological demands, and job insecurity contribute to increases in psychological distress levels. For psychological demands and job insecurity, results accord with those in the literature [9, 11]. Results for skill utilisation levels, however, run counter to tendencies in the literature that show skill utilisation to be negatively associated with psychological distress levels. These results might be explained by the fact that skill utilisation follows a J-shaped nonlinear relationship [19]. Consequently, up to a point, skill utilisation may contribute to lowering psychological distress levels [34]. Beyond a certain threshold, though, continuing pressures to learn new things would constitute an additional workplace constraint [19]. Routine work could, subject to certain limitations, reduce sources of anxiety [24].

Social support in the workplace, as described in the literature [3, 11, 33], helps reduce individual levels of psychological distress. Overall, the workplace, including occupation and working conditions, accounts for 4.3% of the variation in psychological distress over time and 8.3% of the variation among individuals.

The results of our research do not support the second hypothesis (H2), which posits that the relationship between occupation and psychological distress levels is mediated by working conditions. Actually, the results for Model 2 are reproduced in Model 4. Accordingly, even when working conditions are controlled for, white-collar workers and professional workers experience psychological distress levels that are significantly higher than those for the regulated occupations. This means, first, that the impact of one’s occupation on mental health is not transmitted through working conditions and that working conditions in themselves exert an impact on psychological distress, independently from occupation. Nevertheless, it is clear that the low reliability of some scales used by the NPHS could lead to an underestimation of some relationships and therefore impedes our ability to identify the mediation relationship. It is also possible that the working conditions under study do not allow us to identify the mediation relationship. Further studies should be conducted to validate this result, including using more specific working conditions of regulated occupations.

Individual personal characteristics make a major contribution toward explaining psychological distress, alone explaining 17.8% of the variation in distress over time and 34.4% of the variation among individuals. These results accord with earlier research based on NPHS data; those studies arrived at a variation over time close to 20% [20]. The results confirm that several of these characteristics directly influence psychological distress levels (H1), and that this effect remains constant when variables related to the family and the social network outside the workplace are taken into account. The hypothesis that these characteristics moderate the relationship between working conditions and levels of psychological distress receives very little support from our results (H3). For instance, gender (female), smoking, alcohol consumption, and stressful childhood events maintain a direct, positive, significant relationship with psychological distress levels, whereas age, sense of cohesion, and internal locus of control appear to be inversely related to the level of distress. These results are consistent with those found in the literature [40–42, 44, 57, 58].

Bringing in individual personal characteristics, moreover, seems to exert a partial influence on the relationship between occupation and psychological distress. Although the level of psychological distress among white-collar workers appears to be significantly higher than that among professional workers in the regulated occupations (models 2, 4, 6, and 7), this relationship disappears once the personal characteristics of respondents are considered. Other studies should be performed to improve our understanding of how the relationship between occupation and psychological distress might be mediated by personal characteristics.

The third hypothesis (H3) receives only mixed support. Only one interaction between personal characteristics and working conditions—that between social support in the workplace and self-esteem—appears significant. In practical terms, the results we obtained suggest that self-esteem reduces the effect of social support on the level of psychological distress. Social support thus appears to have less influence among individuals with high self-esteem. One hypothesis that could explain this result is the fact that people with strong self-esteem are generally more confident in all areas of their lives, including work. In this context, individuals generally need less social support in the actions of everyday life and the contribution of peer support becomes less important in explaining the level of psychological distress. Conversely, people with low self-esteem need social support when executing such actions because they are insecure. In this case, social support would act as a protective balm for mental health while people with low self-esteem are also generally more likely to experience psychological distress. Further research should be conducted to better understand the dynamics between self-esteem, social support and psychological distress, particularly to confirm whether people with low self-esteem combined with low social support are more vulnerable to psychological distress.

Finally, family characteristics and the social network outside the workplace do not moderate the relationship between working conditions and psychological distress. On the whole, they explain, respectively, 3.6% and 1.7% of the variation in psychological distress over time and 6.4% and 3.0% of the variation in distress between individuals. Moreover, family and the social network outside the workplace do not interact with the workplace to explain psychological distress, which makes confirming H3 impossible. Certain family characteristics, as well as the social network outside the workplace, maintain a direct relationship with psychological distress levels when all dimensions of the model are considered (H1). For instance, being married or in a civil union and having young children (0–5 years old) are significantly and negatively associated with the level of psychological distress, whereas stress in the family setting or in marital or parental relationships is positively associated with the level of psychological distress. The social network outside the workplace, for its part, is significantly and negatively associated with the level of distress, which suggests that such a network has a beneficial effect on individual mental health. Taken together, these results confirm other results published in the literature on the subject [31].

The results we obtained must be interpreted within the limitations of this study. First, we were limited by the indicators used in the NPHS. As a consequence, we were unable to control for variables that could influence the psychological distress of regulated professional workers, such as role conflicts, role ambiguity, and ethical dilemmas. Certain studies, however, have clearly shown that these constraints are linked to psychological distress in some regulated occupations [44, 49, 50]. Nor does the NPHS consider a) certain characteristics of work contracts that are likely to lead to a better work-family balance; b) available occupational health and safety resources; or c) the supervisory styles under which the workers surveyed have been operating. These constitute variables that could well exacerbate or lessen stress experienced in the workplace.

Second, regarding the dependent variable, it should be noted that the main limitation of psychological distress, compared with burnout, is not specific to the workplace, while the intensity of the distress observed may also be the result of other dimensions outside work, in the life of the individual (family or individual characteristics). From a metric point of view, we can still highlight the excellent qualities of the K6 scale to measure psychological distress, which is the scale used by the NPHS. The qualities of this scale, especially with respect to construct validity, have been reiterated recently [70].

Third, the scales that the NPHS used to measure skill utilisation levels, decision authority, psychological demands, and social support in the workplace have lower internal consistency than those used in Karasek's Job Content Questionnaire (JCQ) [63]. This methodological difference could have resulted in underestimating the interactions between these variables and psychological distress levels. The scales measuring decision authority and skill utilisation, however, have been determined to be valid [71]. Nor does the moderate internal consistency of the abbreviated version of the JCQ used in the NPHS pose any major sensitivity problems [72]. Marchand and Blanc [18, 73] validated the reliability over time of the adapted version of Karasek's JCQ [63] used by Statistics Canada. The authors come to the conclusion that repeated measurements of NPHS scales over time have an acceptable reliability, ranging from 0.68 to 0.86, which suggests that the meanings of concept related to these scales have not changed over time [74]. The results obtained by those authors are similar to the reliability obtained on the full version of the JCQ [75].

Fourth, the fact that the NPHS collects data only every two years impedes our ability to grasp the dynamics that affect how individuals experience psychological distress. Major changes may arise unexpectedly in the lives of those surveyed during this period. Such events might well influence psychological distress levels in the workforce.

Fifth, because not all the variables we have selected for study are measured during each survey cycle, we are not in a position to understand fully how they vary over time.

Sixth, this study did not use a simple random sampling design. Because the sampling criteria were not modelled here as levels, we have applied a procedure that consists in correcting the errors by the square root of the general design effect. Even if this strategy is recognised as valid [20, 38, 57], this leads to a 28% increase in the standard errors, which can therefore lead to the conclusion of a non-significant relationship for some variables that had uncorrected p-values ranging from 0.036 to 0.50.

## Conclusion

Despite these limitations, the results we have obtained enable us to add to existing knowledge about the mental health of regulated professional workers, particularly by confirming the existence of a direct link, although relatively weak, between occupation and the experience of psychological distress. Additional research will be required if we are to conduct exhaustive analyses of these occupations in order to explain differences in levels of distress, thereby making it feasible to intervene before mental health problems arise in those categories most severely affected. These results also make clear the importance of developing new tools for measuring psychological distress among upper-level professional workers. First, because traditional models of professional stress were designed when manual labour predominated. Second, because these results highlight the importance of adopting a more dynamic view of stress. Without invalidating traditional models of occupational stress, the latter can capture only part of the dynamics of stressors in the knowledge economy.
